# The Clinical Significance of Blood Examinations in Reference to Surgical Diagnosis and Prognosis

**Published:** 1907-03

**Authors:** Willis Jones

**Affiliations:** Atlanta, Ga.


### ATLANTA
### Journal-Record of Medicine

Successor to Atlanta Medical and Surgical Journal, Established 1855, and Southern Medical Record, Established 1870.

**OWNED BY THE ATLANTA MEDICAL JOURNAL CO.**

**Published Monthly.**

*Official Organ Fnlton County Medical Society, State Examining Board, Presbyterian Hospital, Etc.*

VOL. VIII. MARCH, 1907. No. 12

BERNARD WOLFF, M. D., EDITOR, 319-320 Prudential Bldg.

M. B. HUTCHINS, M. D., BUSINESS MANAGER, 1015-1016 Century Bldg.

E. G. BALLENGER, M. D., ASSOCIATE EDITOR AND ASSISTANT BUSINESS MANAGER.

### ORIGINAL COMMUNICATIONS

### THE CLINICAL SIGNIFICANCE OF BLOOD EXAMINATIONS IN REFERENCE TO SURGICAL DIAGNOSIS AND PROGNOSIS.

By Willis Jones, M. D., Atlanta, Ga.

Since haematology has emerged from a mere theoretical and experimental state, into a science of practical utility in its aid toward more accurate and thorough diagnosis in medical as well as surgical diseases, it will be the purpose of my paper to try and show what has been accomplished along these lines.

As a great majority of the acute conditions which the surgeon is called upon to treat are due to inflammatory changes, and as the blood always plays an important role in these phenomena, it is but natural to suppose that these pathological departures from its normal composition, should be of diagnostic and prognostic aid for long experiment in blood examinations in acute inflammatory conditions has shown that these changes in the blood are fairly constant.

The surgeon of to-day, in taking advantage of these facts, is beginning to rely more and more upon the findings of the clinical laboratory, to aid him in the diagnosis of those cases where the results of bedside observation are not sufficient to warrant positive conclusions. Likewise are blood examinations in post-operative cases very valuable, especially in those cases where shortly after an operation there is a sudden rise of temperature and the operator is baffled to know its origin. In such cases a single blood examination will usually determine at once whether the temperature is due to sepsis or some other condition.

The concensus of opinion of those who are brought daily in contact with inflammatory conditions, where repeated examinations of the blood are made, is that the findings are valuable adjuncts to the surgeon, presenting as they do, both diagnostic and prognostic data, at a time when the clinical picture may be confusing. It gives valuable information concerning the presence or absence of an inflammatory lesion, some idea of its severity and some indication of the resistance the body is offering. In giving consideration to these results, no matter how important, they are not intended to replace clinical and diagnostic skill, nor prognostic ability based on long clinical experience. It is so self evident, as to scarcely need mention, that a blood examination can no more take the place of bedside observations than a urine analysis can indicate the existence of uremic coma without bedside confirmation. Still, this is an argument frequently used against a purpose never intended by the most ardent advocates of the method.

Having given what is considered as sufficient argument for the admission of blood examinations in aiding to make more accurate diagnosis in obscure surgical diseases of inflammatory origin, we are now ready to ascertain what part or parts of the blood's composition will aid in this endeavor. For purely surgical conditions, neither the number of red cells nor the percentage of hæmoglobin are of direct value in determining the character of the disease, for the information obtained, only gives the surgeon an idea of amount of anæmia secondary to the lesion producing the disease. The surgeon, then, for his information, depends upon changes in the white blood cells:

First, Upon the increased number of total white cells present in a given case.

Second, Upon the relative proportions of the polynuclear white cells, for this particular type of cell seems to be one of nature's chief antagonists to septic invasion. It was early determined that in surgical conditions where there was an inflammatory exudate, leucocytosis was found, varying in degree, as a general rule, with the severity of the condition; that a relative increase in the polynuclear leucycuties was an indicator of inflammatory changes, and that the degree of leucycutosis is an evidence of the body resistance toward absorption from these changes. Then it is evident from the above that neither the differential count when considered alone nor the total leucocytyle count when taken alone is of sufficient clinical evidence, and if diagnostic data of material bearing is to be had from the blood examination, it necessarily must be from a careful consideration of the two factors combined. More briefly stated, the total leucocytosis is an index of the body resistance toward infection and must not be looked upon as an indicator of the severity of the pathological process; where as a relative increase in the polynuclear cells is significant of the severity and extent of the lesion, and the greater the disproportion between the total count and the polynuclear per centage the greater is the significance derived therefrom.

In using the results derived from blood examinations to aid in making more positive diagnosis in surgical conditions, we must have some standard for comparison, and, for this purpose, a somewhat higher leucocyte count and polynuclear percentage than the physiological normal is used. Gibson, in his most instructive contribution to this subject in a recent edition of the Annals of Surgery, has established the following arbitrary standard: Commencing with ten thousand leucocytes and seventy per cent polynuclear cells as a basis for comparison, he estimates that for every one thousand increase in the total leucocytes there should be an increase of one per cent. in the polynuclear cells. This standard is purely arbitrary and for purposes of comparison only. He has further found that so long as the line connecting the leucocyte count and the polynuclear percentage is nearly horizontal, that it indicates a lesion; that, whether severe or not, is well borne and therefore, of good prognosis; and that, when a disproportion occurs, that is, the polynuclears increasing more rapidly than the total leucocytes, it indicates in general, a rather severer form of lesion and less body resistance, or both.

When applying this standard of appendicitis studied by him, he found that all the severer lesions, those with gangrene of the appendix or progressive peritonitis, and all his fatal cases showed a rising line or a departure from the horizontal standard, while all the cases that showed a falling line from the horizontal standard were distinctively mild types, such as well defined (safe) abscesses with little febrile or constitutional disturbances. He further states that in the severer cases, particularly those in which the initial symptoms are obscure and perplexing, and when you can least afford to make a mistake, the value of the polynuclear increase is very evident, as the findings in these cases will hardly ever lead you astray.

My own observations, though limited as contrasted with those of Dr. Gibson's, more than confirmed his statements. The standard is not infallible, for in young children, especially infants, the greatest number of disappointments and failures are encountered, for in this class of cases the normal polynuclear percentage is a very variable quantity and low as compared with adults. Other than in children, the findings seem to be practically constant. The essentials of hæmatology, and the practical interpretations of blood examinations, are not too complicated nor too difficult to be mastered by the surgeon, provided he is unable to avail himself of the services of an expert, but this important adjunct to bedside investigation is becoming to be done more and more by the surgeon himself purely for the sake of convenience. Without multiplying instances, we must admit that the examination of the blood has become a part of the equipment of surgical diagnosis, and the surgeon can no more ignore the microscopic diagnosis of neoplasms.

The manufacturers of microscopical instruments, realizing the increasing demand for blood work, have put on the market instruments of such size and construction that they can very easily be carried to the beside of the patient, where all the necessary work can be done.

I do not advocate that the surgeon become dependent for his diagnosis upon the dicta of the laboratory, for if so, he will often find himself at a loss; and too, "he will have robbed himself not only of skill as a diagnostician, but also of the enjoyment of the most interesting feature of his professional work. On the other hand, no surgeon, however great his clinical acumen, however rich his bedside experience, can afford to ignore the exact finding of the microscopist and the pathologist."

319 Century Building.

